# Low temperatures reduce skin healing in the Jacaré do Pantanal (*Caiman yacare*, Daudin 1802)

**DOI:** 10.1242/bio.20135876

**Published:** 2013-09-16

**Authors:** Leandro Nogueira Pressinotti, Ricardo Moraes Borges, Angela Paula Alves De Lima, Victor Manuel Aleixo, Renata Stecca Iunes, João Carlos Shimada Borges, Bruno Cogliati, José Roberto Machado Cunha Da Silva

**Affiliations:** 1 Departamento de Ciências Biológicas/Department of Biological Sciences, Universidade do Estado de Mato Grosso/University of Mato Grosso State, Campus de Cáceres, Avenida São João S/N, Cavalhada Cáceres, MT 78200-000, Brazil; 2 Instituto de Ciências Biomédicas/Institute of Biomedical Sciences, Universidade de São Paulo/University of São Paulo, São Paulo, SP 05508-900, Brazil; 3 Instituto Federal de Mato Grosso/Federal Institute of Mato Grosso, Campus de Cáceres, Cáceres, MT 78200-000, Brazil; 4 Centro Universitário das Faculdades Metropolitanas Unidas (UniFMU)/University Center of the United Metropolitan Universities, São Paulo, SP 01508-010, Brazil; 5 Universidade Paulista (UNIP)/Paulista University, São Paulo, SP 05427-020, Brazil; 6 Departamento de Patologia da Faculdade de Medicina Veterinária e Zootecnia/Department of Pathology of the School of Veterinary Medicine and Animal Science, Universidade de São Paulo/University of São Paulo, São Paulo, SP 05508-900, Brazil; 7 Centro de Biologia Marinha (CEBIMar)/Marine Biology Center, Universidade de São Paulo/University of São Paulo, São Sebastião, SP 11600-000, Brazil

**Keywords:** Crocodilians, Inflammation, Histology

## Abstract

Studies of skin wound healing in crocodilians are necessary given the frequent occurrence of cannibalism in intensive farming systems. Air temperature affects tissue recovery because crocodilians are ectothermic. Therefore, the kinetics of skin wound healing in *Caiman yacare* were examined at temperatures of 33°C and 23°C. Sixteen caiman were selected and divided into two groups of eight maintained at 23°C or 33°C. The studied individuals' scars were photographed after 1, 2, 3, 7, 15 and 30 days of the experimental conditions, and samples were collected for histological processing after 3, 7, 15 and 30 days. Macroscopically, the blood clot (heterophilic granuloma) noticeably remained in place covering the wound longer for the caiman kept at 23°C. Microscopically, the temperature of 23°C slowed epidermal migration and skin repair. Comparatively, new blood vessels, labeled using von Willebrand factor (vWF) antibody staining, were more frequently found in the scars of the 33°C group. The collagen fibers in the dermis were denser in the 33°C treatment. Considering the delayed healing at 23°C, producers are recommended to keep wounded animals at 33°C, especially when tanks are cold, to enable rapid wound closure and better repair of collagen fibers because such lesions tend to compromise the use of their skin as leather.

## Introduction

Crocodilian wound healing and tissue regeneration processes are still poorly studied. There are reports of the tail tip and lower jaw having a partial regenerative capacity. The regenerating vertebral segments are replaced by an endochondral ossification in the form of an unarticulated straight bat. The regenerating skin segment does not repair the osteoderms, therefore differing from the original skin and retaining only pigmentation similar to the original (Alibardi, 2010). There is only one peer-reviewed macroscopic report of that type of regeneration in *Caiman crocodilus* tails (Voigt, 2008).

Several components that may affect the regenerative process have been reported, including innervation, the contact between the epidermis and the lower layers of the skin and muscle layer and, finally, the environmental temperature (Alibardi, 2010). A study of the effect of temperature on the regeneration of the tails of *Anolis carolinensis* lizards indicated that the tail grew longer and faster at 32°C than at 21°C (Maderson and Licht, 1968). Wounds on *Thamnophis sirtalis* snakes healed better at 30°C than at 13.5°C or 21°C as long as the snakes' nutritional demands are met (Smith et al., 1988). To our knowledge, there have been no reports on the effect of temperature on crocodilian wound healing.

Conversely, the effects of temperature on the crocodilian immune system are well studied. Low temperatures tend to decrease the bactericidal, amoebicidal and hemolytic effects of *Alligator mississippiensis* serum (Merchant et al., 2003; Merchant et al., 2004; Merchant et al., 2005); the effect of *A. mississippiensis* phospholipase A2 (Merchant et al., 2009); the hemolytic activity of *Crocodylus porosus* and *Crocodylus johnstoni* sera (Merchant and Britton, 2006) and the activity of dipeptidyl peptidase IV in *Caiman yacare* and *Caiman latirostris* (Siroski et al., 2012). Thus, changes are expected to occur in the wound healing and tissue repair processes of crocodilians subjected to different temperatures.

In other reptiles, including the gecko *Hemidactylus flaviviridis*, temperatures lower than 25°C suppress the activity of phagocytes (Mondal and Rai, 2001). Conversely, in amphibians, the proliferative activity of the lymphocytes and complement system in *Rana pipiens* decrease at 5°C, although there is an increase in the activity of the complement system, above that of the control group, when the frog is transferred from 5°C back to 22°C (Maniero and Carey, 1997). Such a “compensatory” increase was not found in the tail regeneration of *A. carolinensis* lizards when transferred from 21°C back to 32°C (Maderson and Licht, 1968). Following carrageenan challenge and suture, *Rana catesbeiana*, also an amphibian, exhibited a lower number of inflammatory cells in the wound at 6°C than at 24°C (Catão-Dias and Sinhorini, 1999).

Crocodilians' skin provides protection from infection and has commercial value as leather (Woodward et al., 1993). The effect of temperature on skin repair must be examined to aid in avoiding epizootic outbreaks and enable skin repair to maximize its usability as leather, considering the mutilations that frequently occur under farming conditions.

## Materials and Methods

Sixteen *C. yacare* aged between 1 and 1.5 years, donated by the Cooperative of *Caiman yacare* farmers (Cooperativa de Criadores de Jacarés-do-Pantanal (COOCRIJAPAN)) located in the Cáceres municipality, Mato Grosso (MT), were used in this study.

This research was approved by the Commission of Ethics in Animal Experimentation of the Biomedical Science Institute, University of São Paulo (USP), São Paulo (SP).

After intravenously administering anesthesia with propofol at a dose of 5 to 10 mg/kg, animals were subjected to skin abscission, equivalent to the area of an osteoderm (mean area was 91.75±25.13 mm^2^ at dorsal site and 74.80±14.16 mm^2^ at ventral site), in the dorsal and ventral portions of the tail. Subsequently, the caiman were maintained in environments with regulated air temperatures in closed tanks equipped with air conditioning and heaters. Eight animals were maintained in each tank at a temperature of 33±2.0°C or 23±2.0°C. They were fed ad libitum once per day, and the tank was cleaned every 24 hours during the experimental period.

The wounds were photographed 0, 1, 2, 3, 7, 15 and 30 days after the abscission next to a metric scale enabling their measurement. At 3, 7, 15 and 30 days, full lengths of the wounds, until the muscle layer, were collected in two caiman each temperature. The wound samples were divided in half; one half was fixed in McDowell's fixative for 48 h under refrigeration (McDowell and Trump, 1976) for historesin processing for light microscopy and transmission electron microscopy (TEM). The light microscopy preparations were stained using the Rosenfeld method (Rosenfeld, 1947). The other half of each specimen was fixed in Methacarn solution for up to 6 hours and subsequently histologically processed in paraplast for histochemical and immunohistochemical analyses.

Five-micrometer sections were submitted to antigen unmasking in 10 mM citrate buffer solution (pH 6.0) in a pressure cooker for 15 minutes for immunohistochemical labeling. All washings performed as part of that protocol were performed using phosphate buffered saline-tween (PBS-T) solution (pH 7.4). Peroxidase blocking and the nonspecific reaction were performed using peroxidase block (Leica) and protein block (Leica), respectively, for 30 minutes. The antibodies used to assess cell proliferation and identify endothelial cells and pro-angiogenesis cytokine were monoclonal rabbit anti-PCNA (Santa Cruz), polyclonal rabbit anti-Human von Willebrand Factor (Dako) and polyclonal rabbit anti-Human VEGF-A (Santa Cruz), respectively. The antibodies were diluted 1:100 and incubated overnight in a moist chamber. Negative controls were performed omitting primary Ig. Finally, development was performed using post primary block (Leica) for 30 minutes, NovoLink Polymer (Leica) for 30 minutes and the Diaminobenzidine (DAB) precipitation reaction for 45 seconds. The preparations were counterstained with hematoxylin, dehydrated and mounted with coverslips.

Slides with 5-µm-thick sections were embedded in 2% phosphomolybdic acid for 1 minute and stained with picrosirius solution for 1 hour to assess the distribution and direction of collagen fibers. Slides were stained in 1% orcein solution, diluted in 70% alcohol for 30 minutes and counterstained with Harris hematoxylin to identify the elastic fibers in the dermis. Slides were stained using the periodic acid-Schiff (PAS) method to identify the basal lamina region.

Image analyses were performed using Image J freeware, version 1.45c, in 10 pictures distributed of 5 pictures per caiman, in a total of 2 caiman slides at 23°C, 33°C and intact skin. The slides were stained simultaneously to avoid staining variations, and the images were obtained in the same microscopic/camera configuration. The analyses consist of measure and comparison of the areas stained by the picrosirius dye and the staining intensity per pixel between the temperatures and normal skin. Staining intensity per pixel is an inversely proportional value, that is, the brighter pixel was defined as the greatest pixel value (values from 0 to 255) and the less stained the tissue will be, thereby indicating a lower density of collagen fibers. Data were statistically analyzed using *StatSoft STATISTICA 7.0*. The comparisons of collagen area were conducted by nonparametric Kruskal-Wallis test, and the comparison of mean pixel intensity of the picrosirius red-stained area were conducted by one-way ANOVA and the means were compared by Tukey test, respecting assumptions of the Shapiro-Wilk test (normality test) and Bartlett test (homogeneity of variances). Data of the Kruskal-Wallis test and Tukey test were considered significant at *P*<0.01.

## Results

### Macroscopic aspects of tissue wound healing in *Caiman yacare*

**Fig. 1.**
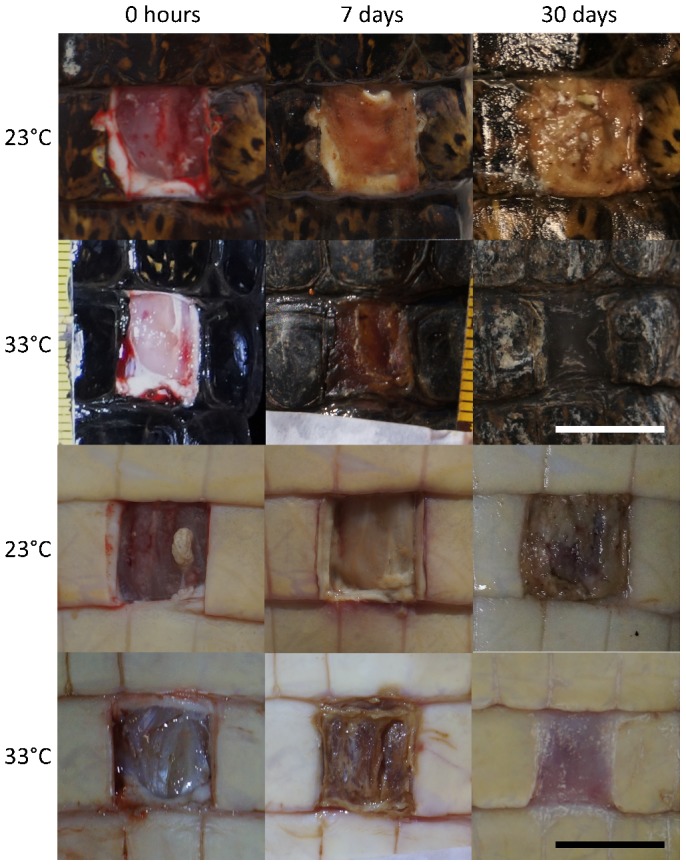
Four series of photographs of the macroscopic view of the healing process of dorsal and ventral wounds in caiman after 0, 7 and 30 days of injury. The pigmented area corresponds to dorsal scar and no pigmented corresponds to ventral scar. It is noteworthy that the wound healed less from day 0 to day 7 at 23°C than at 33°C. Both dorsal and ventral wounds at day 30 at 23°C were still covered by the clot, and their morphology is similar to that of the wounds at day 7 at 33°C. Both dorsal and ventral scar were healed without clot cover at day 30 at 33°C. Scale bars: 1 cm.

The macroscopic analysis of wounds indicated that both dorsal and ventral scars developed similarly at the same temperature. The scars of the individuals raised at 23°C were still translucent at day 7, and they were hidden by a whitish and opaque mass of clotted cells at day 30. The scars at 33°C were hidden by a mass of coagulated cells at day 7 and the blood clot had spontaneously detached itself at day 30, revealing repaired tissue. The dorsal scars were pigmented but the ventral were not (Fig. 1). Thus, caiman maintained at 23°C failed to heal their wounds within 30 days, whereas wound healing occurred at 33°C. The scar size was measured over time and no patterns of wound expansion or contraction were identified over time between temperature treatments, so those data were not included.

### Microscopic morphology of normal skin

**Fig. 2.**
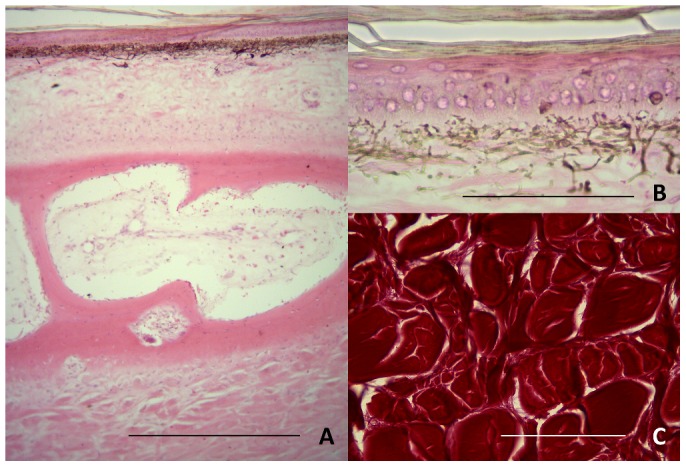
Photomicrographs of normal skin of *C. yacare*. (A) The normal dorsal skin of *C. yacare* are composed by epidermis, lined over loose connective tissue, under the basal lamina region there were many melanocytes. Under the connective tissue an osteoderm can be characterized with medulla. Under the osteoderm, there were the dense connective tissues. (B) High magnification of normal epidermis with 4–6 layers of keratinocytes, striations on lamina basal region, and melanocytes with long projections. (C) High magnification of denser dermis under osteoderm stained by picrossirius, it presents a specific pattern of *raft*, larger fibers interlaced by slim ones. Scale bars: 500 µm (A), 120 µm (B,C).

The dorsal normal skin has an epidermis composed by 4–6 layers of keratinocytes, and there were pronounced striation at lamina basal region and this region was PAS positive. The loose connective tissue was vascularized, with elastic fibers and fibrocytes. Near the vessels there were mast cells characterized under Rosefeld stains. The connective tissue becomes denser at deeper parts until the periosteum of osteoderm. The osteoderm was lamellar with cavities lined by endosteum. The osteoderm medulla had nerves, vascular tissue, and blast cells. Under the osteoderm there was denser connective tissue with fiber disposed in organized pattern similar to a raft, larger fibers interlaced by slim ones (Fig. 2).

### Microscopic general morphology of the wound healing process in *Caiman yacare*

The microscopic descriptions were based on the healing kinetics of the dorsal wound because no significant macroscopic differences were observed between dorsal and ventral wound healing. The below descriptions were valid for animals healing at both temperatures, when not stated otherwise. The wound depth reached the muscle fascia at the site where the osteoderm was collected at day 3 of wound healing. The morphology of the epidermis in the most distant wound areas was similar to that of intact tissue, and only a mild infiltration of heterophils was noted. The heterophils are easily identifiable because they showed eosinophilic granules when stained with Rosenfeld dye. Eosinophilic granules were of variable shapes and sizes, ranging from round to elliptical, and were electron-dense under TEM. In the center of the wound, the damage was covered by an eosinophilic mass of coagulated cells with several visible cell nuclei. Heterophils were the most common clot-forming cells (heterophilic granuloma), and they have preserved morphology in the most basal layers of the clot. Cross sections of muscle fibers were often observed embedded in the heterophilic granuloma.

**Fig. 3.**
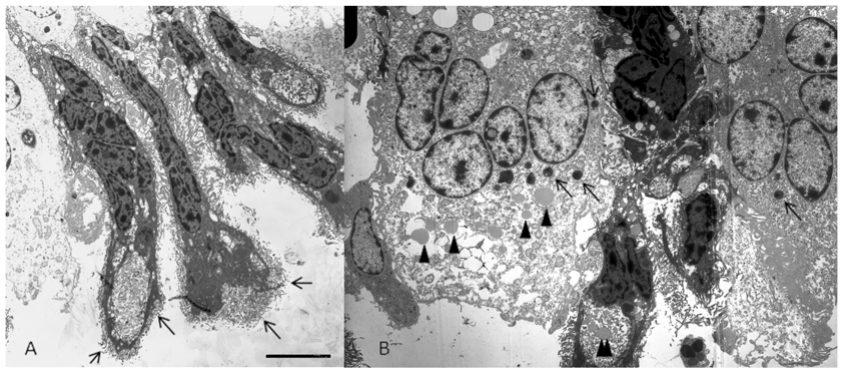
Electron micrograph of wound in *C. yacare* after 15 days of injury at 23°C, giant cells are lined perpendiculary to the heterophilic granuloma at the top and at the bottom continues with the inflammatory loose connective tissue. (A) The electron-dense giant cells with squeezed nuclei, presents many filopodia (arrows) and vesicules filled with amorphous material at the bottom of the cells. (B) Nearby at the same location, electron-lucent giant cells with round nuclei predominantly filled with euchromatin, foamy cytoplasm, granules of heterogeneous electron-density (arrows) and some electron-lucent droplets (arrowhead). An electron-lucent droplet (double arrowhead) within the vesicles of the electron-dense giant cell can be noted in the bottom center of the image. Scale bar: 8 µm.

Rows of multinucleated giant cells forming a palisade layer, delimiting the clot base from the other tissues, were noted below the clot on wounds healing for 3 and 7 days. Ultrastructural analysis confirmed the multinucleated pattern of those cells, with two predominant cell types: giant electron-dense cells and giant electron-lucent cells. The giant electron-dense cells have cell-shaped nuclei, and their outline tends to appear triangular. The cytoplasm has vesicles filled with amorphous material, electron-lucent circular structures and numerous filopodia surrounding the cell (Fig. 3A). The giant electron-lucent cells have round nuclei, predominantly with euchromatin; cytoplasm filled with amorphous membranous compartments, which gives a foamy appearance to cells; and granulation filled with heterogeneous electron-dense content (Fig. 3B). The number of such multinucleated giant cells decreased as the epidermis began to cover the wound. The ultrastructure of macrophages showed nonspecific small granules and few filopodia on the cell surface.

The muscle tissue exhibited intercellular edema and intracellular swelling, identifiable by the distance between myofibrils. It is noteworthy that the nucleus was observed in the center of muscle cells and not at the periphery. The characteristics of inflammation gradually decreased in both the dermis and muscle tissue moving away from the center of the wound until they were undetectable.

Also at day 7, the beginning of an epithelial projection could be observed below the clot. The migrating epidermis had (3–4) few layers, flattened keratinocytes, the basal layer had no striation in basal lamina region, lacked keratin, was thinner than the original and exhibited intercellular edema. The clot increased in thickness and tissue necrosis in the underlying layer was more conspicuous. A greater number of dilated vessels lined with endothelial cells with round nuclei filled with euchromatin were observed in the more distal wound areas.

After day 7, there was a wide variation in morphology between the two temperatures. Notably, new epidermis covered the wound for the individuals maintained at 33°C. For this treatment, there was a clear decrease in the amount of necrosis in the center of the wound. The repaired tissue was basically composed of disorganized thin fibers, fibroblasts and a few inflammatory cells that were still found on the wound. Sometimes, macrophages focus on the formation of giant cells that are able to encircle and remove foreign bodies and newly formed tissue remnants, including fragments of heterophilic granuloma and cellular debris.

### Temperature effect on the microscopic morphology of the wound healing process in *Caiman yacare*

**Fig. 4.**
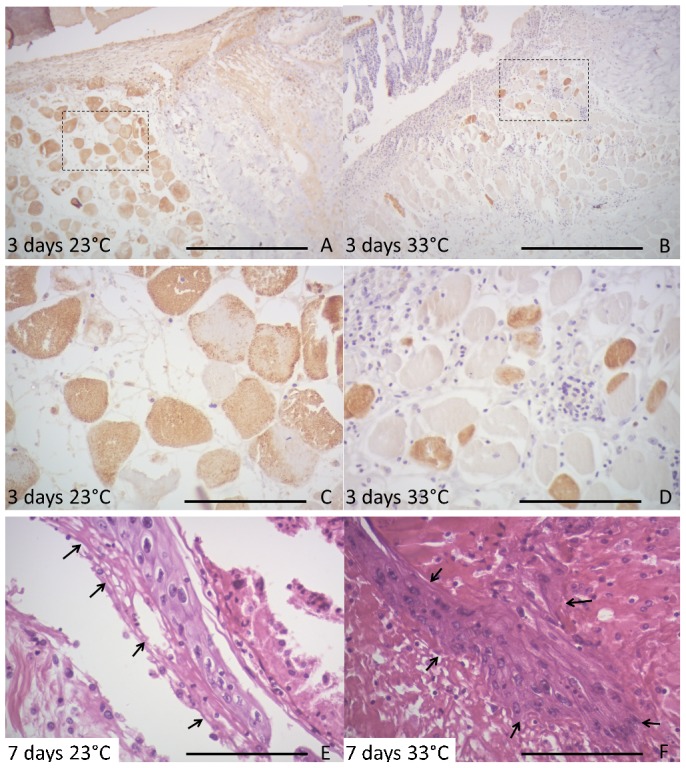
Induced wound in *C. yacare* at both temperatures after 3 and 7 days of injury. The left column shows the scars of caiman maintained at 23°C, whereas the right column at 33°C. The first row shows positive VEGF labeling in inflamed muscular tissue under scab region at 23°C (A) than 33°C (B) at day 3. Panels C and D are of a higher magnification than panels A and B. In panel E, the epidermis (arrows) is detached from the dermis at 23°C, while is attached at 33°C (F). Scale bars: 500 µm (A,B), 120 µm (C–F).

At day 3, the skin wounds of animals maintained at 23°C exhibited a higher detachment of muscle fibers trapped amid the heterophilic granuloma than 33°C. The vascular endothelial growth factor (VEGF) labeling was more frequent in muscle fibers at 23°C than at 33°C (Fig. 4A–D). The muscle fibers under dermis were VEGF positive and frequently had loss their typical form or present edema among myofibrils. Furthermore, the muscle fibers trapped amid the heterophilic granuloma were always VEGF negative. All the immunohistochemistry performed had no labeling at control (suppression of primary Ig) skin sections.

At day 7, discontinuity points of the skin fibers for wounds at 23°C could be noted in all slide sections, this observations were based on the detachment of epidermis from the dermis (Fig. 4E), whereas no detachment were observed at animals healing at 33°C (Fig. 4F). The VEGF labels more muscle fibers at 23°C than at 33°C.

**Fig. 5.**
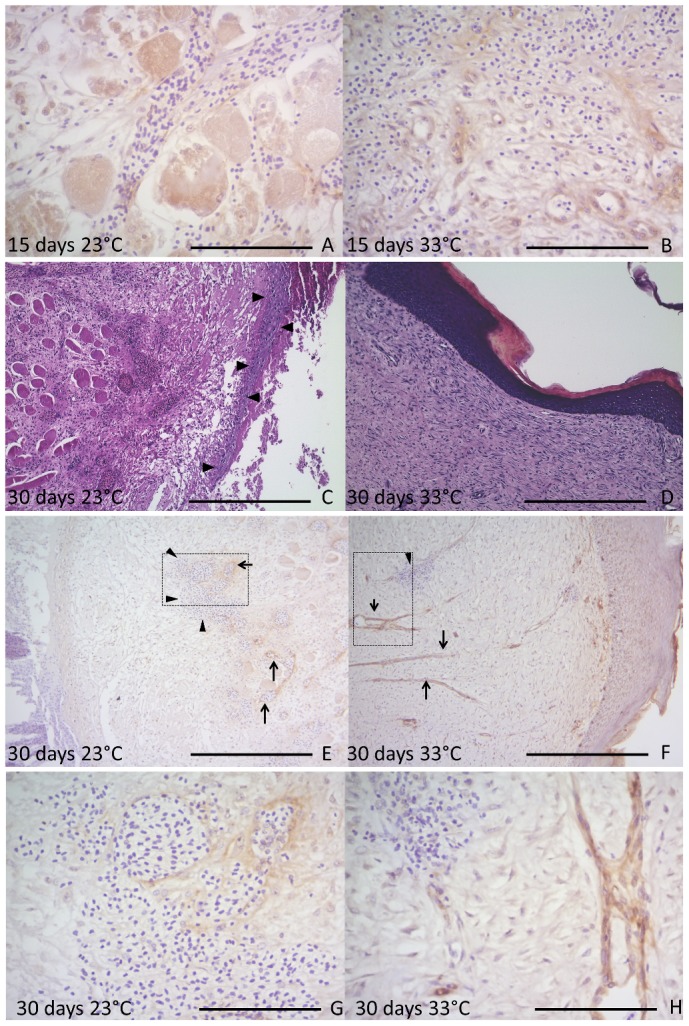
Sequence of induced scars in *C. yacare* after 15 and 30 days. The left column shows 23°C and the right 33°C. (A) The muscular layer and endothelium are vWF positive at 23°C after 15 days. (B) At bottom of the image, the endothelium among fibers is vWF positive, whereas the upper part of the figure there were hemorrhagic among fibers at 33°C day 15. (C) The epidermis (arrowhead) has not completed the migration at 23°C after 30 days. (D) The epidermis recover the normal morphology, with hiperchromic keratinocytes and evident corneal layer at 33°C after 30 days (F). (E) The positively vWF-labeled endothelium shows neovascularization (arrows) and erythrocytes (arrowhead) embedded in the dermis at 23°C day 30. (F) Vessels vWF positive are thinner and perpendicularly positioned between the epidermis and muscle tissue (arrow), and clusters of lymphocytes associated with those vessels are often visible (arrowhead) at 33°C after 30 days. Panels G and H are of a higher magnification than panels E and F, showing endothelium vWF positive and erythrocytes embedded in the dermisin (G). (H) Endothelium vWF positive and a cluster of lymphocytes at upper left of the image. Scale bars: 120 µm (A,B,G,H), 500 µm (C–F).

At day 15, the epidermal migration protrusions still remained detached from the dermis at 23°C, whereas the epidermis at 33°C was observed to be fully repaired. Furthermore, skins of animals healing at 23°C continued to exhibit abundant necrotic material, muscle tissue edema and inflammatory infiltrate, whereas, at 33°C, there were deposition of collagen fibers and the presence of fibroblasts and vessels. The vWF labeling was positive for muscle sections, giant cells, epidermis and endothelial cells and the labeling indicated newly formed vessels in the dermis at 33°C; most of those vessels were congested with erythrocytes. Accumulations of erythrocytes were also found embedded in the matrix but not enveloped by endothelial cells (Fig. 5A,B). The VEGF labels were slight higher in epidermis at 23°C than 33°C, mainly at basal layer of epidermis, and the muscular VEGF labels kept the same differences described above.

**Fig. 6.**
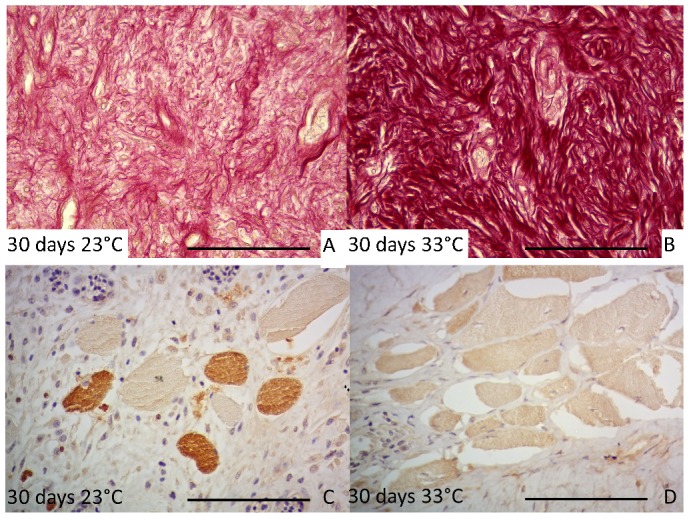
Sequence of induced scars in *C. yacare* after 30 days. The left column shows the scars of at 23°C and the right at 33°C. In the first row, the connective tissue stained by picrosirius is less dense at 23°C (A) than at 33°C (B). In the second row, there is a greater amount of cross-sections of muscle fibers with positive VEGF labeling at 23°C (C) than at 33°C (D). Scale bars: 120 µm.

At day 30 at 23°C, epidermis still did not cover the wound and the mass of necrotic cells and signs of inflammation could still be observed (Fig. 5C). At 33°C dermis was repaired, with a decreased number of vessels congested by erythrocytes, increased blastema and densification of collagen fibers (Fig. 5D). The vWF labeling at 23°C indicated the formation of new skin vessels in an early stage of repair that were frequently congested by erythrocytes, also including accumulations of erythrocytes embedded in the matrix but not enveloped by endothelial cells. At 33°C the skin vessels were thinner, perpendicular to the muscle and epidermal tissues (Fig. 5E–H). Picrosirius staining showed that the density of regenerating skin fibers at 33°C was higher than at 23°C (Fig. 6A,B), indicating that wound healing at 23°C was significantly less fibrous than at 33°C and wound healing at 33°C was significantly less dense than intact skin (Table 1). VEGF labels more muscle fibers at 23°C (Fig. 6C,D), as well as the epidermis at 23°C.

**Table 1.**
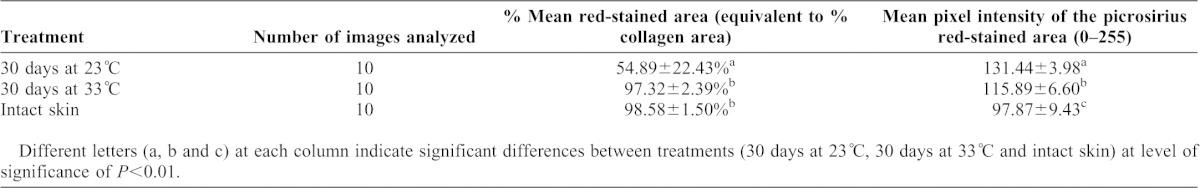
The measurements of picrosirius-stained collagen fibers in the dorsal scars maintained at the two temperatures at day 30.

The proliferating cell nuclear antigen (PCNA) labeling revealed no difference between the two temperatures regarding the distribution of labeled cells; the labeling of nuclei of multinucleated giant cells, blastema cells, muscle fibers and epidermal cells in the basal layer and a few rows above is especially noteworthy. Clusters of PCNA-positive lymphocytes were often observed at 15 and 30 days in the scars maintained at 33°C but not those at 23°C.

Orcein staining did not indicate the presence of elastic fibers in the regenerating tissue, and elastic fibers were identified only in intact tissue located in distal wound areas. PAS staining labeled the basal lamina region only in the outer wound areas, and no staining was found in the basal lamina region using the PAS method, even in regenerated epidermis after 30 days at 33°C. Finally, the muscle tissue with frequent loss of form was PAS-positive in all treatments.

## Discussion

Reptiles are the only ectothermic amniotes and are therefore strategically useful for elucidating the effect of temperature on the immune system (Zimmerman et al., 2010). The results reported herein describe the differences in the histological characteristics of wound healing of *C. yacare* individuals maintained at 23°C and 33°C, revealing a decrease in wound healing ability at the lower temperature.

Macroscopically, there was no pattern of expansion or contraction of the wound edge, and the clear delay in the tissue repair process at 23°C, evidenced by the delay in the shedding of the coagulated mass sealing the wound, is especially noteworthy. This result is consistent with the delay in the regeneration of *A. carolinensis* (Maderson and Licht, 1968), *Podarcis sicula*, *Podarcis muralis*, *Lampropholis delicata*, *Leiolopisma nigriplantare* and *Hoplodactylus maculatum* (Alibardi, 2010) lizard tails and the delay in wound healing of *Thamnophis sirtalis* (Smith et al., 1988) snakes maintained at different temperatures; in all of these instances, a lower temperature was linked to slower regeneration.

Crocodilians with inflammatory processes appear to seek warmer environments, as observed in Lipopolysaccharide (LPS)-challenged *A. mississippiensis*, who engage in search behavior for microenvironments that ensure higher body temperatures (Merchant et al., 2007), temporarily modifying the body temperature comfort zone.

The delay in epidermal migration is microscopically evident at 23°C, a phenomenon previously reported in fish keratinocytes challenged at different temperatures (Ream et al., 2003). The delay in epidermal migration at 23°C resulted in maintaining the heterophilic granuloma on the lesion even after 30 days.

The delay in the formation of new epidermis enables an environment that is conducive to infection. In combination, it is worth noting that the structure of the tanks and the maintenance of caiman with a change of water every 24 hours were not adequate to keep the water free of food debris or waste, making that environment conducive to bacterial proliferation. Furthermore, several mechanisms of the crocodilians' innate immune system are known to decrease in effectiveness with a decrease in temperature (Merchant et al., 2003; Merchant et al., 2004; Merchant et al., 2005; Merchant and Britton, 2006; Merchant et al., 2009; Siroski et al., 2012). At the lower temperature, the wound is exposed for a longer period, increasing the difficulties in keeping the water fully clean, and the immune system activity decreases. All of those factors represent a dangerous combination for farming conditions because they promote infections and decrease the animals' immune defense ability. However, little is known about the variations in *C. yacare*'s immune system with temperature variation (Merchant et al., 2006a; Siroski et al., 2012).

Heterophils act in the re-absorption of residual cellular debris during phagocytosis and defense (Montali, 1988) and are the most abundant crocodilian cells reacting against inflammatory challenges (Merchant et al., 2006b). The presence of extracellular pathogens stimulates the formation of heterophilic granuloma (Montali, 1988). The heterophilic granuloma may contribute to microbicidal activity (Alibardi, 2010), which is corroborated by the lack of infections even after 30 days at 23°C without epidermal cover.

The heterophilic granuloma is frequently encircled by giant macrophage cells, as noted in our findings beginning at 3 days of wound healing, at which point the palisade layers of multinucleated macrophages were observed; corroborating findings have been reported for turpentine-challenged *A. mississippiensis* (Mateo et al., 1984). Rosenfeld staining enabled the recognition of pigments in macrophage vesicles including remnants of heterophils (eosinophilic pigments) and melanin pigments of dark brown color, primarily in dorsal wounds where there is a higher number of pigmented cells, in addition to cell debris.

Vascular endothelial growth factor (VEGF) is part of a family of cytokines that acts in paracrine signaling, enabling healthy angiogenesis and the recovery of tissue functions; their imbalance can result in the formation of granulation tissue and the deregulation of regeneration (Werner and Grose, 2003). The high VEGF immunodetection noted in sections of muscle fibers at 23°C may be related to the persistent inflammatory condition observed that causes the muscular layer to continue to secrete VEGF even after 30 days, as opposed to the lack of VEGF labeling at the muscle fiber trapped amid the heterophilic granuloma, that will be released by the animal. The muscle fiber under the dermis labeled with VEGF indicates that this is actively participating to the inflammatory and proliferative process of wound repair. Furthermore were demonstrated that VEGF is common to be produced by muscle cells during suffering (Rissanen et al., 2002), VEGF diminishes apoptosis in skeletal muscle by autocrine regulation, and participate in proliferation and regeneration of this tissue (Germani et al., 2003; Arsic et al., 2004). Unfortunately the nucleus position was no informative of regeneration in contrast to mammals, once the muscle fibers can have nucleus inside (Claypole, 1897), even in central position, as detected in this work.

Failure to close the epidermis prevents the targeting of new blood vessels because, in addition to perpetuating the inflammatory process, the formation of a VEGF concentration gradient is impaired without an epidermis (Eilken and Adams, 2010). This is in accordance with what was noted at days 15 and 30 days at 33°C, wherein epidermis repaired and the formation of vessels through muscle tissue up to the epidermis were observed, otherwise the epidermis at day 15 at 23°C presented a high VEGF positive labeling at basal layer of migrating epidermis, suggesting communication between dermis and epidermis.

The detachment of epidermis at 23°C reported for days 7 and 15 suggests problems regarding skin repair. Those observations are corroborated by the findings from the use of the picrosirius dye, which stains dermal collagen fibers red. That staining revealed that partial skin repair was only observed after 30 days at 23°C, whereas skin repair was noted beginning at day 15 at 33°C. The repaired skin fibers at 23°C did not correspond to the same pattern of organization of collagen fibers found in the original tissue or in the repaired skin at 33°C. The differences are best evidenced in Table 1, wherein collagen density is significantly different between animals healed at both temperatures and the intact skin control: the smallest marked area and brightest intensity of red pixels, indicating fewer collagen fibers, were found at 23°C, significantly increasing at 33°C and in intact skin. Therefore, the lower temperature not only prevented the wound from closing but also changed the skin's density.

The formation of the extracellular matrix and endothelial cell migration must occur between different stages for angiogenesis to occur (Eilken and Adams, 2010). The difficulty in forming new vessels at 23°C is also related to the lack of an extracellular matrix, as shown by the picrosirius staining. Therefore, temperature affects the extracellular matrix components and the action of blastema cells.

Clusters of lymphocytes infiltrating the skin were observed only in the 33°C group beginning at day 15, always associated with blood vessels. Studies in *Mauremys caspica* turtles showed an increased proliferation ability of lymphocytes in spring, that is, seasons affect lymphocyte proliferation (Muñoz and de la Fuente, 2001). Measurements of thymocyte proliferation in mice were used to estimate the levels of IL-1 expression in LPS-challenged *H. flaviviridis* at different temperatures, finding an optimal temperature of 25°C with a decrease in thymocyte proliferation at temperatures below and above that optimum (Mondal and Rai, 2001). Accordingly, the results of the seasonal effect on lymphocyte proliferation in *M. caspica* may be related to the mean optimal temperature during spring. There are also reports of a decreased lymphocyte proliferative capacity of *R. pipiens* amphibians when maintained at 5°C (Maniero and Carey, 1997), in addition to the aforementioned studies in reptiles. The lacks of clusters of lymphocytes at 23°C is an evidence of the delay in adaptive immune reaction.

The lack of basal lamina, evidenced by PAS technique, under the regenerated epidermis suggests that the barrier between the epidermis and dermis is not complete, allowing a greater exchange of cytokines including VEGF from the epidermis-to-dermis direction. This result is corroborated by the findings of basal lamina discontinuity in *A. carolinensis* visible under TEM (Alibardi, 2010).

The importance of the palisade layer of multinucleated giant cells, muscle fibers and epidermis for regeneration is noteworthy because all of those cells are positively immunolabeled with vWF, VEGF and PCNA, and muscle fibers that are swollen or exhibit a loss of morphological integrity were also stained by PAS. Therefore, the functional plasticity of those tissues deserves further study.

The perpetuation of clusters of erythrocytes embedded in the skin after 30 days at 23°C and the delayed angiogenesis at 23°C may be related to the difficulty of maintaining efficient circulation in the wounded skin, healing at lower temperatures. This possibility is corroborated by observations in *Thamnophis sirtalis* snakes, in which the blood-flow rate tends to decrease and cephalic circulation to increase when maintained at low temperatures because such vascular changes provide greater protection to vital nerve structures when at low temperatures (Amiel et al., 2011). Therefore, low temperatures may compromise not only the rate of reactions but also the intake of cells and molecules required for tail tissue repair and vascular drainage.

The temperature of 23°C not only compromised the set of activities required for tissue repair; it also decreased the animal's appetite, as found in our experiments (data not shown) and reported in studies with snakes (Smith et al., 1988).

Considering the results of this study, it is recommended that when farming *C. yacare* for commercial purposes, wounded caiman should be maintained at 33°C, especially if tanks are cold, enabling a faster epidermal closure and better immune system performance with regard to maintaining the skin integrity (Mitchell and Diaz-Figueroa, 2004). No osteoderms were detected at the repaired skins, and even at 33°C, *C. yacare* skins exhibited a fiber pattern that was significantly different from that of the intact skin, forming scars that may preclude the use of skin as leather; nevertheless, wound healing at 33°C is better recommended than 23°C in terms of skin fiber density.

It is noteworthy that the *C. yacare* farming method in Brazil is a ranching system that includes the regulation of egg collection from wild populations and is authorized by Ordinance no. 126 (Brasil Ministério do Meio Ambiente, 1990). The opportunity for the transfer of infectious agents from wild to captive animals is enhanced at each collection of eggs and/or hatchlings from the wild. Accordingly, optimal conditions enabling organisms to tolerate the entry of those microorganisms must be ensured in the farm at each period of egg collection.

This study is the first to elucidate the effect of temperature on the wound healing ability of *C. yacare*. Furthermore, it supports the conclusion that the temperature of 33°C, the preferred optimal temperature, is best for wound healing. Such findings should be included in discussions regarding the species' management in addition to elucidating histological aspects of the tissue repair process in crocodilians.
